# Polyorchidism in Children: Preserve, Fix, or Remove the Supernumerary Testis?

**DOI:** 10.7759/cureus.117937

**Published:** 2026-10-14

**Authors:** Nikolaos Baltogiannis, Fotini Fili

**Affiliations:** 1 Pediatric Surgery, Children’s Hospital, Athens Medical Group, Athens, GRC

**Keywords:** congenital cryptorchidism, pediatric urology surgery, polyorchidism, supernumerary testis, undescended testis traction orchiopexy

## Abstract

Polyorchidism is a rare congenital anomaly characterized by the presence of more than two testes. Although advances in imaging have improved recognition of supernumerary testes, their optimal management remains controversial, particularly in children. The central therapeutic dilemma is whether a supernumerary testis should be preserved, repositioned and fixed, observed, or removed. Management should be individualized according to testicular viability, morphology, vascularity, anatomical location, relationship with the epididymis and vas deferens, associated cryptorchidism, risk of torsion, oncological suspicion, and the feasibility of long-term surveillance. Available pediatric evidence remains limited and does not allow definitive conclusions regarding long-term malignancy risk, endocrine function, or fertility. In a viable, normal-appearing, and oncologically reassuring supernumerary testis, preservation may be considered when this can be achieved safely, whereas orchiectomy remains appropriate for nonviable, markedly atrophic, dysplastic, or suspicious gonads. Until stronger pediatric evidence and longer follow-up become available, careful anatomical assessment, structured surveillance, and individualized surgical judgment remain essential.

## Editorial

Polyorchidism is sufficiently rare that many pediatric surgeons may encounter only a few cases, if any, during their professional careers. Nevertheless, when an additional testis is unexpectedly identified during investigation or surgery for cryptorchidism, inguinal hernia, torsion, or a presumed inguinal mass, an immediate and potentially irreversible decision may be required: Should the gonad be preserved, repositioned and fixed, observed, biopsied, or removed?

Rather than attempting an exhaustive review of this rare anomaly, this editorial focuses on a specific and practical pediatric surgical dilemma: whether, and under what circumstances, a supernumerary testis should be preserved, fixed, observed, or removed. Our intention is to interpret the available evidence from the perspective of clinical decision-making rather than to provide a comprehensive narrative review of all published cases.

The recent narrative review by Alzahrani et al. provides a broad overview of the clinical manifestations, diagnostic difficulties, malignant potential, and therapeutic considerations associated with polyorchidism [1]. Importantly, it reflects the contemporary transition from historical routine orchiectomy toward a more individualized approach. This shift is particularly relevant in children, in whom preservation of potentially functional gonadal tissue deserves consideration. Nevertheless, because polyorchidism is exceptionally rare and anatomically heterogeneous, and because most available evidence derives from case reports and small retrospective series, rigid therapeutic rules are difficult to justify.

The recent pediatric study by Fang et al. substantially advances this discussion [2]. The authors combined a retrospective two-center pediatric cohort with a systematic review and pooled analysis of published cases. Their findings underline two important issues: diagnostic uncertainty remains clinically relevant, and management strategies vary considerably according to the location and characteristics of the supernumerary testis. Ultrasonography was the principal imaging modality, but diagnostic errors were particularly associated with younger age, extrascrotal location, greater separation from the orthotopic testis, and associated abnormalities [2]. Fang et al. also demonstrated that orchiopexy was more frequently performed than orchiectomy in the pooled pediatric experience and that no malignancy was identified among the available postoperative pathological specimens.

These findings are particularly relevant to pediatric surgical practice. Almost a decade earlier, we emphasized that atypical presentations of a supernumerary testis could lead to misdiagnosis, particularly when the additional gonad presented as an unexpected inguinal structure rather than an easily recognizable third scrotal testis [3]. Therefore, before deciding how a supernumerary testis should be managed, the first priority must be accurate identification of the anatomy.

Ultrasonography with Doppler remains the logical first-line imaging modality when polyorchidism is suspected. It can assess testicular echogenicity, vascularity, size, position, and the relationship between the supernumerary and orthotopic testes. When the anatomy remains uncertain, particularly in an extrascrotal or intra-abdominal location, magnetic resonance imaging may provide additional information. Fang et al. reported higher diagnostic accuracy with magnetic resonance imaging than ultrasonography in their pooled analysis, although the number of magnetic resonance examinations was limited and this observation should therefore be interpreted cautiously [2].

The second question is whether the mere presence of a supernumerary testis constitutes an indication for removal. In our view, it does not automatically do so.

Historical concern regarding malignant transformation contributed substantially to the more aggressive management of polyorchidism. The meta-analysis by Bergholz and Wenke remains an important reference because it demonstrated that anatomical location, drainage pattern, associated cryptorchidism, and malignant potential should all be considered when deciding between observation and surgical treatment [4]. However, assessment of malignancy risk in polyorchidism is particularly difficult. The condition is rare, reported cases are heterogeneous, and long-term follow-up is inconsistent. Furthermore, much of the literature includes both pediatric and adult patients as well as scrotal and undescended testes, making it difficult to determine the independent oncological risk attributable specifically to the presence of a supernumerary testis.

The absence of malignancy in recent pediatric series should therefore not be interpreted as evidence that the long-term risk is negligible. Fang et al. reported no malignant lesions among supernumerary testes for which postoperative pathological findings were available [2], but the number of cases was limited, and follow-up into adulthood was insufficient to establish long-term oncological safety. At the same time, the available evidence does not support assuming that every supernumerary testis carries a degree of malignant risk sufficient to justify prophylactic orchiectomy. The oncological question should instead be considered together with testicular position, morphology, associated cryptorchidism, interval structural changes, and the feasibility of surveillance.

A supernumerary testis may have an epididymal and vasal connection and therefore potential reproductive function. Conversely, some supernumerary testes are markedly hypoplastic or lack an effective drainage pathway. Anatomical classification is consequently useful, but classification should inform management rather than automatically determine it. The relevant clinical questions are whether the gonad is viable, whether it has potentially meaningful function, whether it can remain in or be transferred to a safe and accessible position, and whether its clinical and imaging characteristics are oncologically reassuring.

The pediatric data reported by Fang et al. are particularly relevant in this respect. In their pooled analysis, orchiopexy was the most commonly reported intervention, while orchiectomy was used less frequently and a substantial proportion of children were managed conservatively [2]. These findings suggest that contemporary pediatric practice has already moved away from indiscriminate removal. They should not, however, be interpreted as demonstrating the superiority of preservation, since comparative long-term outcome data are not available.

Our earlier experience with triorchidism associated with cryptorchidism also demonstrated how the anomaly may become apparent only during orchiopexy and how surgical management must be adapted to the actual intraoperative anatomy [5]. Although modern imaging has improved preoperative recognition, the fundamental operative dilemma remains remarkably similar.

For a normally developed, well-vascularized, intrascrotal supernumerary testis without suspicious clinical or imaging characteristics, preservation with surveillance appears to be a reasonable option. In such a setting, removal solely because the gonad is supernumerary may sacrifice potentially functional testicular tissue without a clearly demonstrated benefit. This approach, however, should not be interpreted as a universal recommendation for preservation. The evidence supporting long-term fertility, endocrine, and oncological outcomes remains limited, particularly in children followed from diagnosis through puberty and adulthood.

Management becomes more complex when the supernumerary testis is undescended. Extrascrotal location is associated with greater diagnostic difficulty and may coexist with cryptorchidism or other abnormalities [2]. It may also modify the balance between preservation and removal because an undescended gonad is more difficult to examine clinically and may carry additional risks related to its position. Nevertheless, extrascrotal location alone should not necessarily be regarded as an automatic indication for orchiectomy.

A viable supernumerary testis of reasonable size and morphology, with satisfactory vascularity and without suspicious features, may be considered for preservation and orchiopexy when it can be transferred safely into the scrotum without compromising its vascular supply or that of the orthotopic testis. Conversely, when safe mobilization cannot be achieved, anatomy is severely abnormal, or meaningful surveillance would remain difficult, the threshold for removal may reasonably be lower.

The issue of fixation also deserves attention. Polyorchidism has been associated with testicular torsion, and anatomical abnormalities or increased mobility of an additional gonad may contribute to this risk [1,4]. When surgical exploration is already being performed, and a viable supernumerary testis is preserved, positioning and fixation within the scrotum may therefore be considered when technically feasible. The objective is not merely to preserve testicular tissue, but to leave it in an anatomically safe and clinically accessible position that facilitates subsequent examination and imaging.

Surveillance of a preserved supernumerary testis should also be considered part of the management decision rather than an afterthought. Because no evidence-based pediatric surveillance schedule has been established, follow-up must currently be individualized. A practical approach may include periodic clinical examination and ultrasonography with Doppler to document testicular position, size, echotexture, morphology, and vascularity and to identify interval changes. The frequency of imaging should be adapted to the patient's age, testicular location, associated abnormalities, and initial imaging findings rather than prescribed as a universal fixed interval.

Particular attention should be given during pubertal development, when changes in testicular volume and symmetry may provide additional information regarding gonadal development. New pain, enlargement, a palpable abnormality, altered consistency, or suspicious ultrasonographic changes should prompt earlier reassessment and, when appropriate, additional imaging or surgical evaluation. Tumor markers or more advanced imaging should be guided by clinical suspicion rather than used routinely in every asymptomatic child with reassuring findings.

The limitations of surveillance must nevertheless be acknowledged. There are insufficient long-term data to determine whether preserved supernumerary testes contribute meaningfully to fertility, whether their endocrine function differs from that of orthotopic testes, or whether lifelong oncological risk differs according to anatomical subtype. Long-term pediatric cohorts extending through puberty and into adulthood are particularly lacking. Consequently, recommendations regarding preservation and surveillance remain based on incomplete evidence and should be discussed with patients and families accordingly.

Conversely, orchiectomy remains appropriate when the potential benefit of preservation is minimal or when preservation introduces disproportionate risk. A clearly nonviable testis, a markedly atrophic or dysplastic gonad, progressive structural abnormality, suspicious heterogeneous morphology, or a lesion in which malignancy cannot reasonably be excluded represents a different clinical situation. In such circumstances, preservation of gonadal tissue should not take precedence over oncological safety.

A practical pediatric surgical framework may therefore be based on four questions: Is the supernumerary testis viable? Is it potentially functional? Can it remain or be positioned safely within the scrotum? Is there credible oncological concern? When viability, favorable anatomy, safe positioning, and reassuring clinical and imaging characteristics coexist, preservation can reasonably be considered. When one or more of these conditions are absent, the balance may shift toward orchiectomy.

Fang et al. should be commended for attempting to transform a fragmented body of pediatric case-based literature into a practical diagnostic and therapeutic framework [2]. Their proposed algorithm is particularly valuable because it acknowledges the importance of location, associated cryptorchidism, torsion, imaging findings, and other complications. At the same time, the authors appropriately recognize that the framework remains based largely on retrospective and case-derived evidence rather than prospectively validated clinical data [2].

This limitation is fundamental. The rarity of polyorchidism makes randomized trials unrealistic, but prospective multicenter registries would be achievable and clinically valuable. Future studies should systematically document anatomical location, testicular dimensions, vascularity, epididymal and vasal relationships, operative findings, histopathology when available, pubertal development, endocrine function, fertility-related outcomes, and long-term oncological surveillance. Standardized reporting would also help determine whether outcomes differ between scrotal and extrascrotal testes and between gonads with and without reproductive ductal connections.

Until stronger prospective pediatric evidence and longer follow-up become available, management should remain individualized. A viable supernumerary testis should not be removed solely because it is supernumerary. Preservation may be appropriate when the gonad is anatomically satisfactory, well vascularized, potentially functional, safely positioned or repositionable, and without suspicious clinical or imaging findings. Orchiopexy or fixation may be considered when safe scrotal positioning can reduce mechanical risk and facilitate surveillance. Orchiectomy remains appropriate for nonviable, markedly atrophic or dysplastic, technically unsuitable, or genuinely suspicious gonads.

In pediatric polyorchidism, therefore, the objective should not be to establish preservation or removal as a universal rule. The more appropriate goal is to identify which supernumerary testes can be safely and meaningfully preserved and which cannot. At present, careful anatomical assessment, cautious interpretation of the limited evidence, practical long-term surveillance, and individualized surgical judgment offer the most defensible approach.
